# A 4D Theoretical Framework for Measuring Topic-Specific Influence on Twitter: Development and Usability Study on Dietary Sodium Tweets

**DOI:** 10.2196/45897

**Published:** 2023-06-13

**Authors:** Lingchao Mao, Emily Chu, Jinghong Gu, Tao Hu, Bryan J Weiner, Yanfang Su

**Affiliations:** 1 Milton Stewart School of Industrial and Systems Engineering Georgia Institute of Technology Atlanta, GA United States; 2 Interlake High School Bellevue, WA United States; 3 Department of Economics University of Washington Seattle, WA United States; 4 Department of Geography Oklahoma State University Stillwater, OK United States; 5 Department of Global Health University of Washington Seattle, WA United States

**Keywords:** social media, health education, health promotion, dissemination strategy, influence, Twitter, activity, priority, originality, popularity

## Abstract

**Background:**

Social media has emerged as a prominent approach for health education and promotion. However, it is challenging to understand how to best promote health-related information on social media platforms such as Twitter. Despite commercial tools and prior studies attempting to analyze influence, there is a gap to fill in developing a publicly accessible and consolidated framework to measure influence and analyze dissemination strategies.

**Objective:**

We aimed to develop a theoretical framework to measure topic-specific user influence on Twitter and to examine its usability by analyzing dietary sodium tweets to support public health agencies in improving their dissemination strategies.

**Methods:**

We designed a consolidated framework for measuring influence that can capture topic-specific tweeting behaviors. The core of the framework is a summary indicator of influence decomposable into 4 dimensions: activity, priority, originality, and popularity. These measures can be easily visualized and efficiently computed for any Twitter account without the need for private access. We demonstrated the proposed methods by using a case study on dietary sodium tweets with sampled stakeholders and then compared the framework with a traditional measure of influence.

**Results:**

More than half a million dietary sodium tweets from 2006 to 2022 were retrieved for 16 US domestic and international stakeholders in 4 categories, that is, public agencies, academic institutions, professional associations, and experts. We discovered that World Health Organization, American Heart Association, Food and Agriculture Organization of the United Nations (UN-FAO), and World Action on Salt (WASH) were the top 4 sodium influencers in the sample. Each had different strengths and weaknesses in their dissemination strategies, and 2 stakeholders with similar overall influence, that is, UN-FAO and WASH, could have significantly different tweeting patterns. In addition, we identified exemplars in each dimension of influence. Regarding tweeting activity, a dedicated expert published more sodium tweets than any organization in the sample in the past 16 years. In terms of priority, WASH had more than half of its tweets dedicated to sodium. UN-FAO had both the highest proportion of original sodium tweets and posted the most popular sodium tweets among all sampled stakeholders. Regardless of excellence in 1 dimension, the 4 most influential stakeholders excelled in at least 2 out of 4 dimensions of influence.

**Conclusions:**

Our findings demonstrate that our method not only aligned with a traditional measure of influence but also advanced influence analysis by analyzing the 4 dimensions that contribute to topic-specific influence. This consolidated framework provides quantifiable measures for public health entities to understand their bottleneck of influence and refine their social media campaign strategies. Our framework can be applied to improve the dissemination of other health topics as well as assist policy makers and public campaign experts to maximize population impact.

## Introduction

### Background

Health promotion is important for updating knowledge, attitudes, and behaviors regarding essential health topics among individuals, households, and communities. However, it is challenging to understand which way of promotion works and which does not and how to promote health-related information outside of traditional approaches such as radio broadcasting, television, newspapers, and magazines. The percentage of US adults who use at least one social media tool has grown from 5% in 2005 to 72% in 2020 [1]. Social media can play a promising role in reducing the know-do gap between research and practice and in linking health practitioners, policy makers, and funding agencies with the public [1]. Social media make health communication more convenient and complex, as it is inexpensive, timely, interactive, and reaches dynamic audiences.

Twitter has become one of the largest social networking services in the world since its launch in 2006. Twitter has been extensively used by government agencies, nongovernment organizations, academic organizations, and experts for health information dissemination [2]. For example, Twitter has been used to bring awareness of childhood obesity [3], promote smoking cessation [4], or communicate desired health behaviors during a pandemic [5,6]. Nevertheless, previous studies suggested that public health organizations need to better harness Twitter’s potential to improve their public reach and attention on Twitter [4,7].

To optimize public influence on Twitter, it is important to quantitatively evaluate different dissemination strategies. However, there is no consensus yet on how to quantify a user’s influence on Twitter. An influencer is not necessarily the one creating the most popular tweets or amassing the highest number of followers. There is a broad categorization of influencers based on different behavior patterns. Influencers include but are not limited to authoritative policy makers, domain experts, and opinion leaders [8]. Opinion leaders can be further categorized into idea starters, amplifiers, curators, commentators, and viewers [8]. An effective measure of influence should differentiate influencer type and provide tailored insights to improve information dissemination. Additionally, influence is topic-specific. Studies in implementation science show that clinical opinion leaders are generally monomorphic, that is, exercising influence only in their areas of expertise, and specialized opinion leaders are more likely to lead effective dissemination strategies [9,10]. A recent analysis of the World Health Organization (WHO)’s tweets revealed that diffusion size, a measurement of tweet spread, varied based on the topic [11]. For example, the WHO’s tweets on viruses demonstrated more widespread diffusion than tweets on physical activity [11]. Thus, measures of influence need to not only consider influencer type but also be topic-specific.

Commercial tools have been developed to analyze the social media engagement of Twitter accounts. Currently active commercial tools include Socioviz, Tweet Binder, Social Bearing, Hootsuite Insights, Followerwonk, Twitonomy, Audiense, Keyhole, and Meltwater (see the list and each tool’s functionalities in Multimedia Appendix 1). Most commercial tools provide influencer identification capabilities but are copyright protected and require paid subscriptions [12]. One of the few exceptions is Socioviz [12], a social network analysis tool that measures user influence by the numbers of retweets and mentions received. However, the free version of Socioviz is only able to offer analyses for tweets posted a week prior and only supports 1 search slot for a keyword, user, etc. More sophisticated commercial tools analyze nonpublic metrics such as the number of impressions and number of clicks; however, these metrics can only be obtained for an access-granted account. These limitations restrain commercial tools from being used on a larger scale.

After the launch of the Twitter full-archive search application programming interface (API) in 2015 [13], accessing Twitter data for public use has become convenient. Compared to that in commercial tools, there is a gap to fill in advancing publicly accessible social media analysis tools with transparent computations that can be applied to any Twitter account. To address this gap, we reviewed existing measures of influence from literature to propose a consolidated framework capable of quantifying topic-specific influence on Twitter for public use.

### Related Work

Many measures have been proposed to quantify influence on social media. Note that the definition of influence on social media is slightly different from the notion of influence in other interactions. Although one could assess changes in people’s awareness, knowledge, attitudes, or intentions, it is more common in social media research and practice to assess influence in terms of engagement with social media content. We followed the categorization in Riquelme et al [8] to synthesize existing measures of influence, activity, and popularity.

Influence, sometimes called authority, measures the degree of reach to other users in the Twitter network. One approach of social network analysis is to construct a graphical representation of Twitter data and then measure the accessibility of each node (user) with respect to other nodes (users) in the network. Existing methods in this category differ in how edges between nodes are defined (eg, based on account relationships, retweets, mentions) and the centrality measure employed, such as closeness, betweenness, H-index, and eigenvector centrality [14]. Several measures are based on PageRank [15], a well-known algorithm developed in 1998 to measure the relative importance of websites on the internet, such as TunkRank [16], Influence Rank [17], SpreadRank [18], and Author-Reader Influence [19]. Some studies further considered the propagation of tweets through the network, differentiating direct and indirect influence [20]. However, these graph-based centrality measures can be costly due to the time consumed for each data request in Twitter and the computational complexity of large networks [8].

Activity refers to a user’s participation in a social network. Examples of activity measures include TweetRank (total number of tweets by the user), Tweet count score (number of original tweets and retweets), and general activity (sum of visible actions of a user) [8]. To measure the activity related to a specific topic, algorithms such as Topical Signal and Signal Strength [7] have been proposed by previous studies.

Popularity refers to the attention a user receives from the social network. A convenient proxy for popularity is the number of followers in Twitter and its variants, such as the follower-following ratio (relative number of followers versus following) and follower rank (proportion of followers out of the sum of followers and following) [21]. However, measures based on account relationships ignore the tweet-level data and cannot be used to analyze topic-specific impact. Other measures such as acquaintance-affinity score [8] and action-reaction [22] leverage tweet-level data such as likes, retweets, and mentions to measure public attention with additional granularity.

### Objective

This research aims to develop a consolidated framework to measure topic-specific user influence on Twitter. We first specified our working definitions for influence, activity, priority, originality, and popularity. Then, we proposed a framework that decomposes a summary indicator of influence into 4 measures—activity, priority, originality, and popularity—to characterize the different tweeting dissemination strategies and emphasize topic-specific influence to tailor dissemination strategies on topics of interest. We demonstrated this framework by using a case study of dietary sodium tweets. The aim of this case study was to analyze the influence of 16 organizations or individuals on the topic of dietary sodium, quantify their tweeting patterns, and provide personalized recommendations for the promotion of sodium-related content. Unlike existing commercial tools that are copyright protected and targeted toward private access–granted Twitter accounts, our framework can efficiently measure public influence for any Twitter account based on publicly accessible information provided by the Twitter API. We aim to develop a generic framework intended for public use and for providing support to public health agencies to improve their dissemination strategies. More importantly, our framework can be easily applied to various applications with social purposes.

## Methods

In this section, we first describe the development of a 4D framework to measure topic-specific influence and then perform a usability study of dietary sodium tweets. Diseases related to high dietary sodium consumption are cardiovascular diseases, as high sodium intake is a risk factor for cardiovascular diseases. The sample population in this study consisted of 33 Twitter accounts from June 30, 2006, to May 31, 2022, with data accessible through Twitter API v2.

### Summary Indicator of Influence

Our working definition of topic-specific influence refers to the capacity of an individual or entity to engage the public on Twitter within a specific subject area. The degree of engagement between the influencer and their audience is assessed by the cumulative number of topic-related interactions generated through content creation over a given period. In Twitter, public engagement is captured by 4 public metrics: number of likes, quotes, replies, and retweets. Thus, we compute total tweet-level public engagement as the sum of the number of likes, quotes, replies, and retweets. At the user level, the summary indicator of topic-specific influence is the total public engagement received from all tweets that are relevant to the topic of interest. We emphasize that this summary indicator can be computed with public metrics from Twitter API without the need for private access. Considering N as the number of tweets, R as the number of retweets, P as the number of public engagements, and superscript t as topic-specific, the summary indicator of influence can be calculated as the product of 4 measures:

Influence_topic_ = Activity · Priority · Originality_topic_ · Popularity_topic_ = N · N^t^/N · (N^t^ – R^t^) / N^t^ · (P^t^_like_ + P^t^_reply_ + P^t^_quote_ + P^t^_retweet_) / (N^t^ – R^t^) = P^t^_like_ + P^t^_reply_ + P^t^_quote_ + P^t^_retweet_ = Total topic-specific public engagements

**Figure 1:**
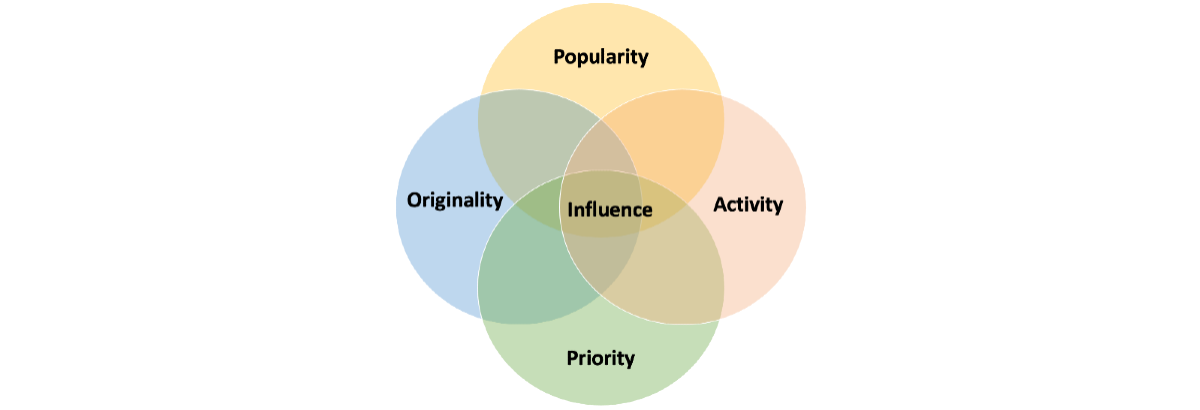
The 4D framework for measuring influence.

In the next section, we discuss these 4 dimensions of influence in detail. An overview of the framework is shown in Figure 1. This mathematical linkage provides the summary indicator with a granular breakdown of tweeting patterns to derive personalized topic-specific dissemination strategies.

### 4D Measures of Influence

An overview of the 4 dimensions of influence is shown in Table 1.

**Table 1:** Summary of the 4D measures of influence.^a^

Dimension	Description	Measure	Level
Activity	Level of participation on Twitter	N	Account level
Priority	Relative importance of a given topic within the scope of work	N^t^/N	Account level
Originality	Proportion of tweets that required creation of original content	(N^t^ – R^t^) / N^t^	Account level
Popularity	Average number of public engagements received per content-creation tweet	(P^t^_like_ + P^t^_reply_ + P^t^_quote_ + P^t^_retweet_) / (N^t^ – R^t^)	Account level

^a^N is the number of tweets, R is the number of retweets, P is the number of public engagements, and superscript t means the metric is topic-specific.

### Activity

In our study, activity is defined as a user’s level of effort on Twitter. Taking into consideration commonly used activity measures such as TweetRank (total number of tweets of the user) [8,23], Tweet count score (number of original tweets and retweets) [8], and General Activity (sum of visible actions of a user) [23,24], we used the total number of tweets, N, as our activity measure because this measurement is often easy to obtain:

Activity = N

### Priority

Existing studies of Twitter either measured general influence or topic-specific influence. We establish a relationship between general tweets and topic-specific tweets called as priority. Priority is defined as the percentage of topic-specific tweets (N^t^) out of the user’s total number of tweets (N): 

Priority = N^t^/N.

Intuitively, priority measures the relative importance of a given topic within the organization or individual’s scope of work. Although less discussed in the literature, we argue that this measure is an important driver of tweeting patterns and should be considered in dissemination strategies.

### Originality

It is important to differentiate different types of tweets in Twitter, as each type has a different interaction dynamic. Twitter offers 4 types of tweets: original tweet, quote, reply, and retweet (see definitions in Multimedia Appendix 2). Since the first 3 types of tweets involve the creation of new content, we categorized them as content-creation tweets. A retweet solely republishes a tweet and is the only type of tweet without public metrics (ie, number of likes, quotes, retweets, and replies) collected by Twitter. Instead, the public interactions gained from a retweet are accumulated toward the original tweet. This backend design of Twitter fully captures the public metrics of original content, but credits retweets toward the original content creators instead of disseminators. We define the originality measure as the proportion of content-creation tweets over the total number of tweets. The topic-specific originality is computed as:

Originality_topic_ = (N^t^ – R^t^) / N^t^

### Popularity

Popularity is measured by the amount of public attention that an organization or individual receives on Twitter. Twitter defines engagement as the “total number of times a user interacted with a tweet. Clicks anywhere on the tweet, including retweets, replies, follows, likes, links, cards, hashtags, embedded media, username, profile photo, or tweet expansion” [25]. Four of these engagement types, that is, likes, retweets, replies, and quotes, are publicly retrievable from the Twitter API and are known as public metrics, whereas others are stored as private metrics. Since our goal is to derive an easy-to-access evaluation tool, we only used publicly available metrics to measure public engagement. At the tweet level, we summed the total count of likes (P_like_), quotes (P_quote_), replies (P_reply_), and retweets (P_retweet_) as the total public engagement of each tweet. At the user level, we measured popularity as the average number of public engagements per content-creation tweet. The topic-specific popularity is computed as:

Popularity_topic_ = (P^t^_like_ + P^t^_reply_ + P^t^_quote_ + P^t^_retweet_) / (N^t^ – R^t^)

Note that retweets were excluded from this computation because public metrics are not collected for retweets.

### Stakeholder Selection

We selected 8 pairs of domestic and international stakeholders with scopes of work relevant to dietary sodium. This selection included public agencies, academic institutions, professional associations, and domain experts. A total of 33 Twitter accounts from 16 organizations and individuals were analyzed (Table 2). Note that some organizations had more than 1 Twitter account relevant to dietary sodium, and data were aggregated at the stakeholder level. The selection of the 16 organizations might introduce sampling bias. The purpose of this case study was to highlight the proposed framework rather than to provide an exhaustive assessment of tweeting behaviors for representative sodium-related organizations on Twitter.

**Table 2:** Sixteen stakeholders related to dietary sodium tweets and their 33 associated Twitter accounts.^a^

Category, scope, organization/individual				Twitter handle
**Public agencies**				
	**Domestic**			
		Centers for Disease Control and Prevention (CDC)	CDCgov CDCDirector CDCFound CDC_eHealth	
		United States Department of Agriculture (USDA)	USDA USDANutrition TeamNutrition NationalCACFP SNAP_Ed BeAFoodHero NatWICAssoc	
	**International**			
		Food and Agriculture Organization of the United Nations (UN-FAO)	FAO	
		World Health Organization (WHO)	WHO	
**Research and evaluation organizations**				
	**Domestic**			
		Harvard University (Harvard)	HarvardChanSPH HarvardHealth HSPHnutrition Harvardmed	
		Stanford University (Stanford)	StanfordMed SJPHonline	
	**International**			
		University of Oxford (Oxford)	NDMOxford UniofOxford	
		University College London (UCL)	UCLeHealth BScPopHealth	
**Professional and advocacy associations**				
	**Domestic**			
		American Heart Association (AHA)	American_Heart AHAScience	
		Center for Science in the Public Interest (CSPI)	CSPI	
	**International**			
		World Heart Federation	worldheartfed	
		World Action on Salt (WASH)	WASHSALT actiononsalt	
**Experts**				
	**Domestic**			
		Kirsten Bibbins-Domingo	KBibbinsDomingo	
		Tom Frieden	DrTomFrieden	
	**International**			
		Simon Capewell	SimonCapewell99	
		Alexey Kulikov	KulikovUNIATF	

^a^Four stakeholders were chosen from each of the 4 categories: public agencies, research and evaluation organizations, professional and advocacy associations, and experts. Half of the stakeholders had domestic scope and the other half had international scope.

### Data Collection

Data were retrospectively collected from the 33 Twitter accounts from June 30, 2006, to May 31, 2022, via Twitter API v2. The Academic Research license enabled us to carry out a full-archive retrieval of every tweet since Twitter’s conception. We retrieved tweet-level data, including creation date, author username, full tweet text, language, public metrics (number of retweets, quotes, likes, and replies), mentions, and hashtags. The account-level data retrieved included username, display name, creation date, location, number of total tweets, number of followers, and number of followings (retrieval date September 30, 2022). The tweets from multiple accounts of the same stakeholder were concatenated into 1 data set. The definitions of Twitter-specific terminology used in this study can be found in Multimedia Appendix 2.

A keyword-based algorithm was used to determine whether a tweet was dietary sodium–related. The algorithm first removed content involving English slangs that contained the keyword “salt.” See Multimedia Appendix 3 for a list of 24 English slangs obtained from The Free Dictionary [26]. Then, the slang-free content was flagged as sodium-related if at least one of the following criteria was met: (1) contained keywords (“sodium” OR “salt”) OR ((“salty” OR “salts” OR “salted”) AND (food-related keyword OR health*)) in the tweet, where the asterisk means any group of characters, or (2) used hashtags related to dietary sodium (Multimedia Appendix 3). The list of food-related keywords was compiled from synonyms of “food” and “eating” from the English Thesaurus [27] (Multimedia Appendix 3).

### Ethical Considerations

Since this is a second-hand data analysis with publicly available data, it is exempted in terms of institutional review board review. Data used in this study received explicit consent for research purposes from Twitter. Twitter users have the right to make changes to the availability of their public content, such as tweet addition, deletion, and other user-driven changes. Users of Twitter data respect these changes and only access data that has current public disposition. It is also worth noting that this study only used summary statistics such as total counts of tweets and public measures without analyzing tweet content in detail.

## Results

### Sample Characteristics

**Figure 2:**
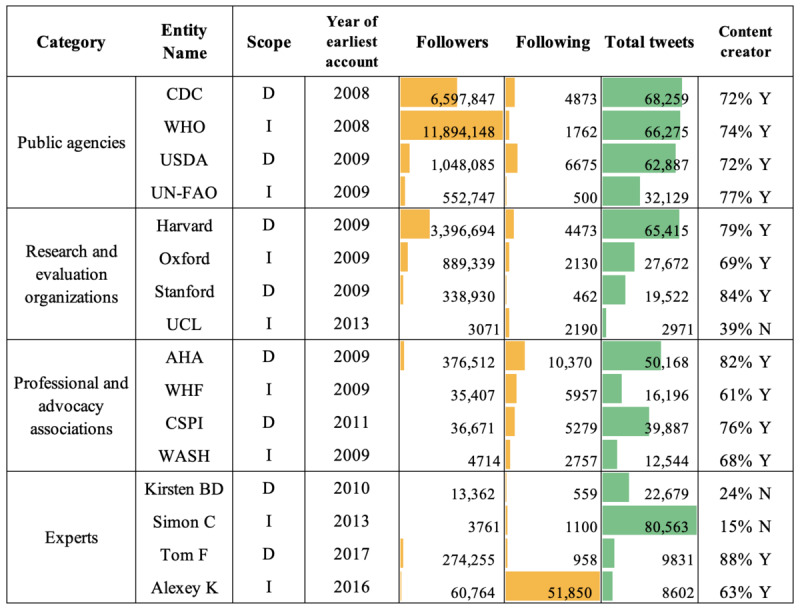
Stakeholder-level characteristics of data collected from 33 Twitter accounts of 16 stakeholders from June 30, 2006, to May 31, 2022, through the Twitter application programming interface v2. Green bar: number of tweets; Yellow bar: number of followers; American Heart Association; CDC: Centers for Disease Control and Prevention; CSPI: Center for Science in the Public Interest; D: domestic; I: international; N: no; UCL: University College London; UN-FAO: Food and Agriculture Organization of the United Nations; USDA: United States Department of Agriculture; WASH: World Action on Salt; WHF: World Heart Federation; WHO: World Health Organization; Y: yes.

A total of 585,600 tweets were retrieved from the 16 stakeholder accounts, of which 12,393 were considered sodium-related. Figure 2 shows an overview of the Twitter data of the stakeholders, including the year the earliest account was created, number of followers and following, total number of tweets, and the percentage of content-creation tweets. An example of the Twitter interface with user-level data, tweet-level data, and different public engagements can be found in Multimedia Appendix 4. A stakeholder was considered a disseminator if more than 50% of their tweets were retweets. Most stakeholders actively tweeted original content, except for University College London, Kirsten Bibbins-Domingo, and Simon Capewell, whose activities were dominated by retweets. As expected, large organizations such as WHO, Centers for Disease Control and Prevention (CDC), United States Department of Agriculture, and Harvard University had the largest number of followers and were well-established macroinfluencers in the public health area. Note that 2 stakeholders, Tom Frieden and Alexey Kulikov, joined Twitter relatively recently compared to other stakeholders. Multimedia Appendix 5 shows the tweets with the highest number of public engagements from each stakeholder as an example of their contribution.

### Influence Analysis

**Figure 3:**
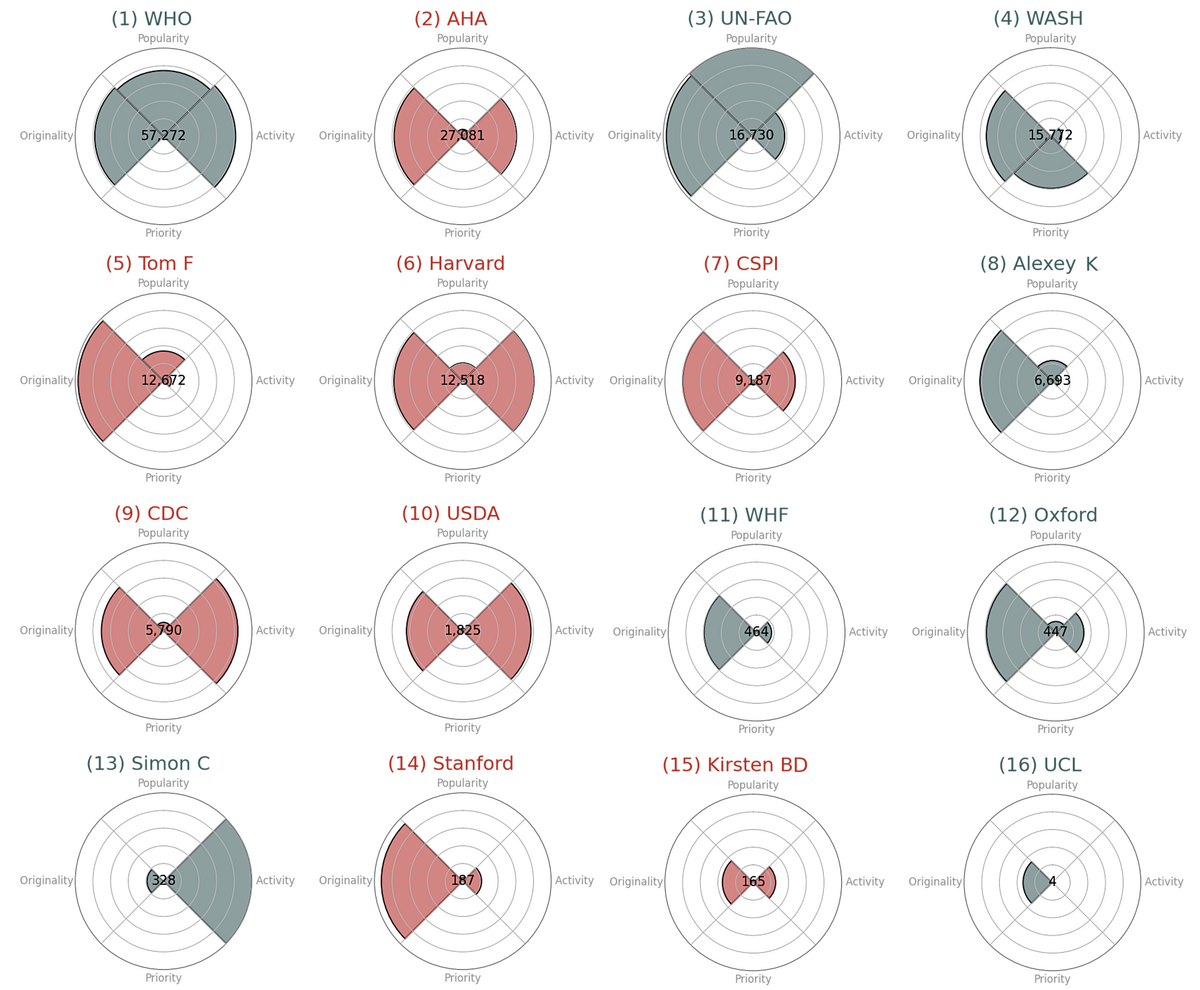
Influence and its contributing 4 dimensions calculated for each of the 16 stakeholders by using Twitter data collected from June 30, 2006, to May 31, 2022; 16 stakeholders sorted in the descending order by the summary metric of influence (in the bull's eye of the target). Color coding indicates whether the stakeholder is domestic (red) or international (grey). Priority and originality ranged from 0 to 1. Popularity ranged from 3 to 299 (minimum and maximum values within the sample). Activity ranged from 2971 to 80,563 (minimum and maximum values within the sample). AHA: American Heart Association; CDC: Centers for Disease Control and Prevention; CSPI: Center for Science in the Public Interest; UCL: University College London; UN-FAO: Food and Agriculture Organization of the United Nations; USDA: United States Department of Agriculture; WASH: World Action on Salt; WHF: World Heart Federation; WHO: World Health Organization.

The 4D analysis of influence for each stakeholder is shown in Figure 3. With a given set of stakeholders, the summary indicator of influence can be used to rank stakeholders based on their overall topic-specific influence. For instance, in our example, WHO had the largest influence in sodium-related tweets according to the summary metric, followed by American Heart Association (AHA), Food and Agriculture Organization of the United Nations (UN-FAO), and World Action on Salt (WASH).

As hypothesized, the top influencers in this sample exhibited different tweeting patterns. WHO’s high influence was due to its high performance in 3 out of 4 dimensions: large tweeting volume, high portion of original content, and high popularity. AHA, Harvard, Center for Science in the Public Interest, CDC, and United States Department of Agriculture had a similar pattern for activity and originality but low level of priority and popularity. In contrast, Tom Frieden and Alexey Kulikov were low-volume content creators but still influential due to their high popularity. While having a similar overall influence as UN-FAO, WASH had a noticeably different influence profile with the highest priority in creating sodium content but the lowest popularity in this sample. At the lower end of this spectrum, Oxford, Stanford, and World Heart Federation had low influence in dietary sodium due to relatively low performance in 3 out of 4 dimensions, that is, low activity, priority, and popularity. Simon Capewell, Kirsten Bibbins-Domingo, and University College London also had low influence measures with regard to sodium.

### Group Analysis of Influence

**Figure 4:**
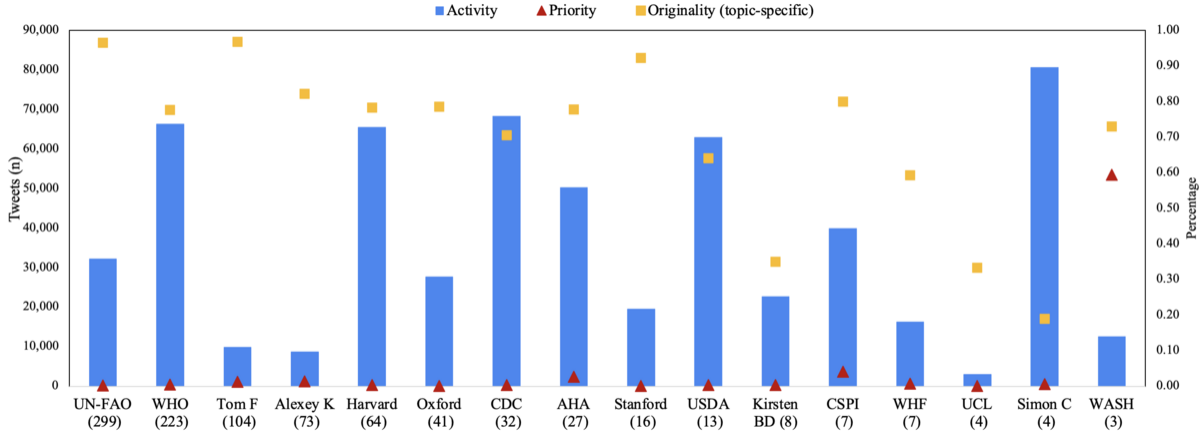
Comparisons across stakeholders by their activity, priority, topic-specific originality, and topic-specific popularity. The left-hand y-axis represents the number of tweets, which is the scale for activity (blue bars). The right-hand y-axis represents percentage, which is the scale for priority and topic-specific originality. All stakeholders in the x-axis were sorted by topic-specific popularity (in parenthesis). AHA: American Heart Association; CDC: Centers for Disease Control and Prevention; CSPI: Center for Science in the Public Interest; UCL: University College London; UN-FAO: Food and Agriculture Organization of the United Nations; USDA: United States Department of Agriculture; WASH: World Action on Salt; WHF: World Heart Federation; WHO: World Health Organization.

Figure 4 compares the stakeholders with respect to each of the 4 dimensions. In terms of activity, 1 individual, that is, Simon Capewell, had the highest total tweet volume since Twitter’s inception. The majority of Simon Capewell’s tweets were retweets, which involved less time commitment compared to tweets that involve content creation. WHO, Harvard, CDC, AHA, and United States Department of Agriculture had a similar activity level but notably different levels of popularity. This comparison reinforces the hypothesis that an effective dissemination strategy should not only focus on increasing tweet volume but also other dimensions of influence.

In terms of priority, most sampled stakeholders had very low priority for dietary sodium in their communications on Twitter (<0.5%), showing that sodium was not a popular dissemination topic in these large health organizations. WASH, an international group, focused on reducing salt and sugar intake and had, by far, the highest priority for sodium (7462/12,544, 59.5%), followed by Center for Science in the Public Interest (1630/39,887, 4.1%) and AHA (1312/50,168, 2.6%).

In terms of originality, UN-FAO, Tom Frieden, and Stanford were the most dedicated content creators. Although content creation is traditionally seen as a priority in information dissemination and the design of Twitter’s metrics also favors content creation, Simon Capewell showed as an exception. Despite having the lowest originality level in this sample and less followers, Simon Capewell still had a higher influence measure than Stanford. This case shows that retweeting can be a relatively time-efficient approach to increase influence and boost public engagement of the content-creation tweets.

In terms of popularity, UN-FAO, WHO, and Tom Frieden had the highest popularity, with more than 100 public engagements per sodium-related content-creation tweet on average. However, WASH had the lowest popularity in this sample despite having the highest priority for dietary sodium. Two of the top 3 stakeholders in popularity were not the ones with the highest number of followers, which reflects that the number of followers does not necessarily indicate topic-specific popularity. Note that Tom Frieden and Alexey Kulikov were among the stakeholders with the highest popularity for sodium-related tweets despite joining Twitter relatively late, that is, in 2017 and 2016, respectively.

### Comparison With Traditional Measure of Influence

A common measure of influence used by commercial tools as well as in literature is the number of followers, which is a publicly available measure that can be conveniently obtained without computations. We conducted a statistical comparison of our summary indicator of influence with this traditional measure of influence. Since the number of followers is clearly non-Gaussian in our sample (ie, some macroinfluencers have a larger number of followers), we used a nonparametric correlation test, Kendall rank correlation test, which does not require distribution assumptions. The Kendall rank correlation coefficient between the summary indicator of influence and number of followers for this stakeholder sample was 0.742 (*P<*.001). These results suggest that the proposed summary indicator is notably aligned with this traditional measure of influence. Note that the main driver of this association is the popularity measure, as users with more followers have more public exposure and thus tend to receive more public engagement per tweet. Our summary indicator of influence is a more granular measure than the number of followers, as it captures tweet-level public engagement and topic-specific influence.

## Discussion

### Principal Findings

We proposed a consolidated multidimensional framework to measure topic-specific influence on Twitter. This framework not only allows ranking of users based on their summary indicator of topic-specific influence but also is a decomposable characterization of their influence into 4 measures—activity, priority, originality, and popularity. We demonstrated the proposed framework by using a case study on dietary sodium with a sample of 16 stakeholders and more than half a million tweets to analyze their sodium-specific influence on Twitter.

This framework has several innovations. The decomposability is a key improvement over traditional single-dimensional measures, allowing visibility into different determinants of influence. This property is important, as we showed in the case study that stakeholders with a similar level of influence, such as WASH and UN-FAO, had similar overall influence but notably different tweeting patterns and hence are suited for different dissemination strategies. Our summary indicator of influence was statistically correlated with a traditional measure of influence, that is, number of followers, and can measure topic-specific and tweet-level public engagement that solely the number of followers fails to capture. Additionally, we introduced the concept of priority as a measure of relative importance of the topic within the organization or individual’s scope of work. Taking dietary sodium as an example, WHO was a macroinfluencer with a high level of activity and originality but low priority for dietary sodium in its scope of work. In contrast, microinfluencers such as WASH had a high priority for sodium content. We also emphasized originality to differentiate content creation from retweets. For Twitter accounts with significant efforts toward retweeting, for example, Simon Capewell, retweets still indirectly boosted influence even as their public engagement was credited toward another account’s tweet. Thus, retweeting can be a relatively time-efficient approach to increase influence.

This framework can be used from 2 perspectives. For an organization or individual whose goal is to increase their influence on Twitter, this framework can be used to evaluate their current presence on Twitter and to identify strengths and weaknesses. To achieve high influence on a given topic, an organization needs to be competent in multiple dimensions of influence. Take the sodium case as an example: only the top influencer, WHO, was high performing in 3 of the 4 dimensions of influence, while other health organizations and individuals had room for improvement in at least 2 of the 4 dimensions. WASH had the highest priority for sodium content but needed to focus on improving popularity, that is, average engagement per content-creation tweet. To increase popularity, strategies suggested by previous studies include inviting macroinfluencer accounts to retweet or engage with their tweets or framing tweet content in ways that evoke emotion and demonstrate usefulness [28]. The design of the specific strategy should be consistent with the culture, climate, and resources of the organization [29]. We refer interested readers to Purtle et al’s [30] research for more lessons regarding better dissemination of public health content to practice. In summary, an organization should invest in improving their weakest dimension to achieve optimal growth in overall influence.

For public health initiatives or campaigns, this framework can be used to compare the tweeting patterns of a given list of organizations and identify the most appropriate influencers to promote the topic of interest. Influencers who have low topic-specific originality, such as Simon Capewell in the sodium case study, should be noted as promising disseminators, as they are likely to retweet sodium-related content created by other users. Further, the priority dimension can be viewed as a probability of engagement with the influencer. For example, while macroinfluencers such as WHO and UN-FAO have a much wider public reach on Twitter than smaller institutions, dietary sodium was low priority in their current scope of work compared to other health topics. The priority measure suggests that the probability of engaging WHO and UN-FAO in a sodium reduction campaign may be lower than that of engaging institutions with a higher priority measure for sodium.

### Limitations

This work has several limitations. First, similar to other existing measures based on public metrics, our popularity measure cannot be computed for retweets, as public metrics for retweets are counted toward the original tweet. Hence, we do not have the ability to measure the influence of retweets themselves, which is an important dissemination medium, beyond simply counting the number of retweets. Second, our popularity calculation equally weighs the 4 types of public engagements—likes, quotes, replies, and retweets. One could argue that these engagements require different levels of time commitment and consequently have varying impacts on the original tweet’s dissemination. Therefore, further research can explore assigning different weights to public engagements. Third, we utilized all content-creation tweets, including original tweets, replies, and quotes, to compute the originality measure and to compare against retweets. Determining the first appearance of content on Twitter is beyond the scope of this work. Fourth, our keyword-based criteria to determine sodium-related tweets in a big data archive may include noisy data and may be limiting for more abstract or complex topics. Employing human labelers or advanced machine learning models may provide more accurate topic labeling in those cases. Note that detailed tweet-level content analysis (eg, content subcategories, media attachment, writing style, sentiment analysis) was outside the scope of this work. Lastly, our case study only focused on tweets in English, while international organizations such as WHO publish a small portion of tweets in other languages.

### Conclusions

This study lays out efficient and accessible methods to measure user influence for any Twitter account and given topic of interest. The proposed framework can be used to identify influencers and characterize their distinctive dissemination strategies on Twitter, accompanied by a quantifiable visualization of influence with its 4 dimensions: activity, priority, originality, and popularity. We illustrated this framework by using a case study on dietary sodium by evaluating 16 health domain stakeholders’ influence. The case study showed that each stakeholder had different strengths and weaknesses in their dissemination strategies, and 2 stakeholders with similar overall influence could adopt significantly different tweeting patterns. Our framework can be applied to improve the dissemination of other health topics by enhancing influence visibility and forming tailored recommendations to optimize user’s time and financial investments on health education and promotion on Twitter. These capabilities can assist public health entities and policymakers in refining their social media campaign strategies on Twitter to maximize their population impact.

## Appendix

Multimedia Appendix 1 Currently active commercial media analysis tools and functionalities.

Multimedia Appendix 2 Key terminology and features of Twitter.

Multimedia Appendix 3 List of English slang terms, dietary sodium-related hashtags, and food-related keywords.

Multimedia Appendix 4 Examples of Twitter account-level interface, tweet-level interface, and different public engagements.

Multimedia Appendix 5 Top tweets with the most public engagements from each stakeholder.

## Abbreviations

AHA: American Heart Association
API: application programming interface
CDC: Centers for Disease Control and Prevention
UN-FAO: Food and Agriculture Organization of the United Nations
WASH: World Action on Salt
WHO: World Health Organization

## Data Availability

The data sets analyzed during this study are available with Twitter's Academic access and approval from Twitter. More details about Twitter’s access tiers and their monthly limits of data retrieval can be found in Twitter API access guidelines [31].
