# Supplementary material for: A 4D Theoretical Framework for Measuring Topic-Specific Influence on Twitter: Development and Usability Study on Dietary Sodium Tweets
Source: J Med Internet Res. 2023 Jun 13;25:e45897. doi: 10.2196/45897 (PMC10337429; doi:10.2196/45897)
Supplement: Multimedia Appendix 1 [file jmir_v25i1e45897_app1.pdf]

**Analytical tool**	**Identification of influential users**	**Price**	**Link**
Socioviz	Yes. User-level influence is determined by the numbers of retweets and mentions received. Supports keyword-based search.	Free version, then academic version 49€/year, business version 199€/year.	https://socioviz.net/
Tweet Binder	Yes. User-level influence is determined by the product of number of tweets and number of followers. Supports keyword-based search.	$80-$1300/month, or a $60-$240 one-off payment. 6-day free version.	https://www.tweetbinder.com/
Social Bearing	Yes. User-level influence determined by "reach" defined as the number of followers from each unique user or retweeting user. Supports keyword-based search.	7-day free trial, premium services currently unavailable	https://socialbearing.com/
Hootsuite Insights	No. Focuses social media marketing for a private account and targeted for commercial use (e.g., ads management).	30-day free trial, then ranges from $49/mo to $739/mo	https://www.hootsuite.com/
Followerwonk	No. Focuses on social media marketing for a private account for commercial use. Does not support topic-specific analysis.	Free version with one profile, then from $29/mo to $79/mo	https://followerwonk.com/
Twitonomy	Yes. User-level influence determined by the number of followers. User-level engagement measured by the number of retweets and favorites generated by their tweets mentioning the keyword.	Free version, then premium is $20 single, $19/mo, and $199/year	https://www.twitonomy.com
Audiense	No. Focuses on social media marketing for a private account for commercial use.	Free version, then £32/mo to £556/mo	https://www.audiense.com/
Keyhole	Yes. Influential users identified using keywords and hashtag tracking analytics. Overall engagement rate, reach, and impressions are considered.	Free trial, then $39/mo to $79/mo	https://keyhole.co/
Meltwater	Yes. Influential users identified using keyword and hashtag tracking analytics and evaluated based on number of posts, reach, clicks, and social Return on Investment.	Price based on quote	https://www.meltwater.com/en
