# Supplementary material for: A 4D Theoretical Framework for Measuring Topic-Specific Influence on Twitter: Development and Usability Study on Dietary Sodium Tweets
Source: J Med Internet Res. 2023 Jun 13;25:e45897. doi: 10.2196/45897 (PMC10337429; doi:10.2196/45897)
Supplement: Multimedia Appendix 2 [file jmir_v25i1e45897_app2.pdf]

Feature	Description
Tweet	A message posted to Twitter containing text, photos, a GIF, and/or video. It appears on the author’s profile page.
Reply	A tweet posted in direct response to another user’s tweet, starting with the “@” symbol proceeded by the replied username. The user can optionally reply to multiple users in a conversation thread. The user being replied receives a notification directing them to the reply tweet. It appears on the author’s profile page.
Retweet	A tweet republished by another user. The original author receives notification that their tweet is retweeted. It appears on the author’s profile page.
Quote	A retweet with comment. It appears on the author’s profile page.
Mention	A tweet text containing another user’s username, which is preceded by the “@” symbol. The users being mentioned receive a notification directing them to the tweet.
Like	An action button below each tweet represented by a small heart to show appreciation for a tweet.
Hashtag	Written with a # symbol, hashtags are used to index keywords or topics.
Followers	A list of users that subscribe to a user’s account to receive notification about their Twitter activities.
Following	A list of users that a user subscribes to receive notification about their activities.
