# Supplementary material for: A 4D Theoretical Framework for Measuring Topic-Specific Influence on Twitter: Development and Usability Study on Dietary Sodium Tweets
Source: J Med Internet Res. 2023 Jun 13;25:e45897. doi: 10.2196/45897 (PMC10337429; doi:10.2196/45897)
Supplement: Multimedia Appendix 3 [file jmir_v25i1e45897_app3.pdf]

List of English slangs

'pinch of salt', 'dash of salt', 'worth their salt', 'worth his salt', 'worth her salt', 'worth my salt', 'worth your salt', 'grain of salt', 'attic salt', 'eat their salt', 'dose of the salts', 'salt into the wound', 'salt in a wound salt down', 'salt away', 'salt horse', 'salt of the earth', 'salt the books', 'salt-and-pepper', 'above the salt', 'back to the salt mines', 'below the salt', 'pound salt', 'old salt'

List of curated dietary sodium-related hashtags

'switchthesalt', 'breakupwithsalt', 'escapethesalt', 'hiddensalt', 'noaddedsalt', 'nosaltplease', 'nycsalt', 'reducesalt', 'restrictsalt', 'saltintake', 'saltsubsitute', 'saltsubstitutes','saltsugfat', 'saltswitch','shakethesalthabit', 'sneakysalt', 'saltweek2018', 'stopsaltstopstroke', 'stopthehiddensalt', 'toomuchsalt','uksaltchallenge', 'unsalted', 'spotthesalt','WASHSALT','cashsalt' ‘*lesssalt*”, “*lessalt*”, “*lowsalt*”, “*highsalt*”, “*lowsodum*”, “*highsodium*”, “*saltawareness*”,”*saltreduction*”

List of food-related keywords:

'bite', 'board', 'bread', 'breakfast', 'butter', 'chewing', 'chow', 'consumption', 'cookery', 'cooking', 'cuisine', 'diet', 'dinner', 'drink', 'eat', 'eating', 'eats', 'entrée', 'fare', 'fast food', 'feasting on', 'feeding on', 'food', 'foodstuff', 'gobbling', 'goodies', 'gorging on', 'groceries', 'grub', 'handout', 'having a meal', 'health', 'home cooking', 'intake', 'lunch', 'luncheon', 'meal', 'meat', 'munching', 'nibbling', 'nutrition', 'overindulgence', 'pigging out', 'ration', 'snack', 'snacking', 'stuffing oneself', 'sustenance', 'table', 'take out'
