# Supplementary material for: A 4D Theoretical Framework for Measuring Topic-Specific Influence on Twitter: Development and Usability Study on Dietary Sodium Tweets
Source: J Med Internet Res. 2023 Jun 13;25:e45897. doi: 10.2196/45897 (PMC10337429; doi:10.2196/45897)
Supplement: Multimedia Appendix 4 [file jmir_v25i1e45897_app4.pptx]

## Slide 1
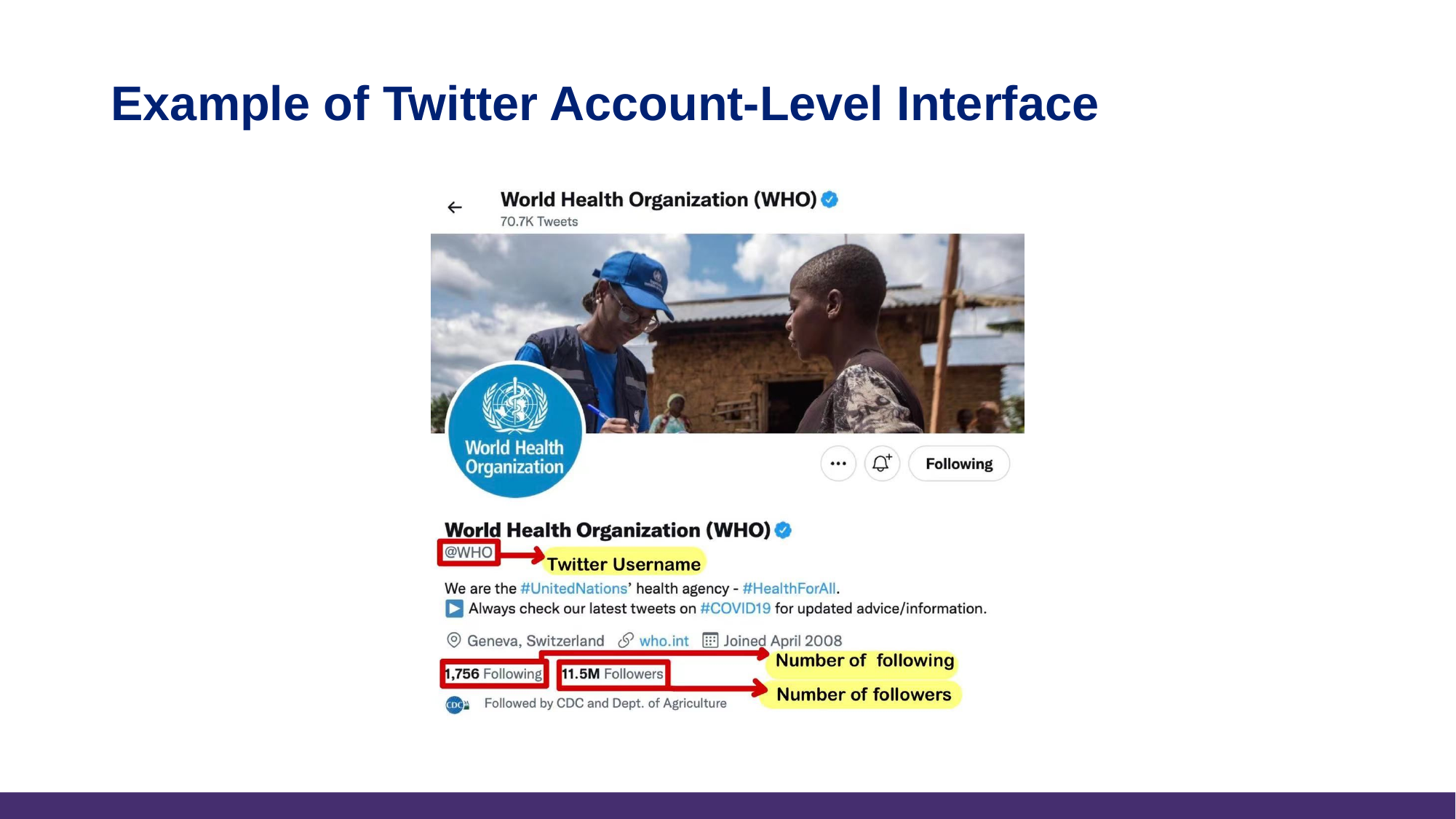

# Example of Twitter Account-Level Interface

## Slide 2
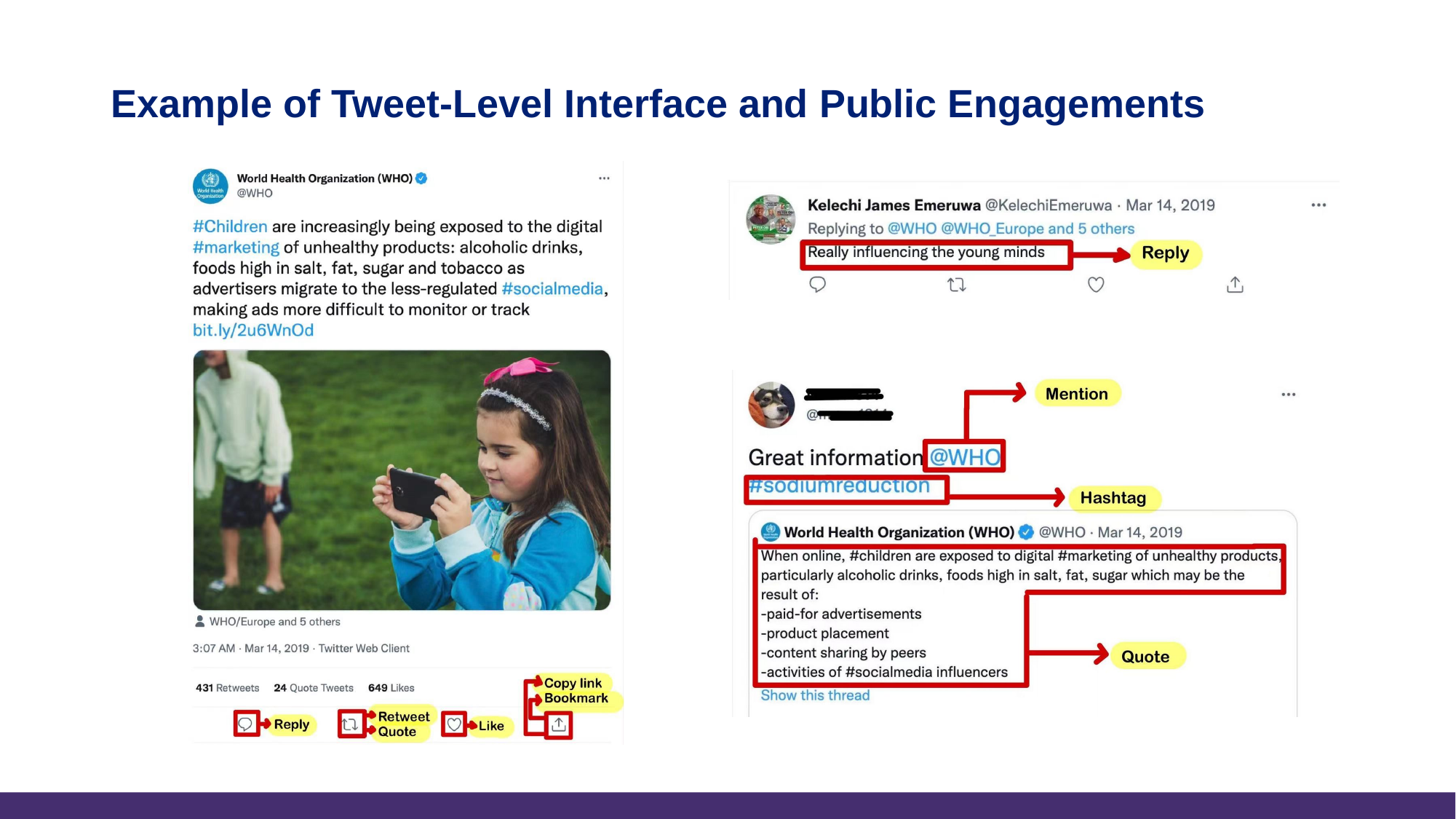

# Example of Tweet-Level Interface and Public Engagements
