# Supplementary material for: A 4D Theoretical Framework for Measuring Topic-Specific Influence on Twitter: Development and Usability Study on Dietary Sodium Tweets
Source: J Med Internet Res. 2023 Jun 13;25:e45897. doi: 10.2196/45897 (PMC10337429; doi:10.2196/45897)
Supplement: Multimedia Appendix 5 [file jmir_v25i1e45897_app5.pdf]

Top tweets with most public engagements from each stakeholder:

Author (handle)	Created date	Text	Public engagement
American Heart Association (American_Heart)	May 5, 2016	Cheering @Nestle for supporting release of voluntary #sodium standards. #BreakUpwithSalt RT if you agree!	*total*=809<br>*retweets*=237<br>*replies*=29<br>*likes*=442<br>*quotes*=0
Alexey K (KulikovUNIATF)	September 24, 2020	Strong message from @DrTedros<br>“The tragedy is that much of #NCDs suffering is preventable. And we know what the answers are:”<br>🚭 Stop tobacco use<br>🍺 Reduce harmful use of alcohol<br>🧂 Cut salt intake<br>🧁 Consume less sugar<br>🏃 ♀ Increase physical activity	*total*=282<br>*retweets*=85<br>*replies*=12<br>*likes*=175<br>*quotes*=10
Centers for Disease Control and Prevention (CDCgov)	October 13, 2021	High sodium intake contributes to high rates of high blood pressure, heart attack, & stroke. ↓ in sodium intake could result in tens of thousands fewer cases of heart disease and stroke each year.<br>@US_FDA	*total*=456<br>*retweets*=113<br>*replies*=42<br>*likes*=284<br>*quotes*=17
Center for Science in the Public Interest (CSPI)	April 4, 2016	Over time, the food industry has increased the amounts of sugar, salt, and fat in our food. #NotOurChoice	*total*=70<br>*retweets*=42<br>*replies*=2<br>*likes*=24<br>*quotes*=0
Harvard University (HarvardChanSPH)	June 11, 2019	Our researchers estimate that:<br>- Scaling up high blood pressure treatment could extend the lives of 39.4 million people<br>- Cutting sodium intake by 30% could stave off 40 million deaths<br>- Eliminating trans fat could prevent 14.8 million early deaths	*total*=860<br>*retweets*=358<br>*replies*=11<br>*likes*=465<br>*quotes*=26
Kirsten Bibbins-Domingo (KbibbinsDomingo)	October 15, 2021	Thrilled @US_FDA setting dietary sodium targets<br>Our 2010 @NEJM paper found even modest reductions ➡️ substantial health & economic benefits<br>▶️ All segments of US population benefit<br>▶️ cost-saving<br>▶️ magnitude of benefits on par w other public health targets	*total*=48<br>*retweets*=11<br>*replies*=1<br>*likes*=35<br>*quotes*=1
University of Oxford (UniofOxford)	March 30, 2019	People in rich countries consume too much fat, sugar and salt, and we can expect obesity to rise in other countries as they abandon traditional foods. Susan Jebb, @OxPrimaryCare, explains how legislative powers can be used to improve the food system:	*total*=80<br>*retweets*=26<br>*replies*=3<br>*likes*=48<br>*quotes*=3
Simon Capewell (SimonCapewell99)	April 19, 2018	#JunkFood exposed!<br>7.30pm tonight<br>by @EmmaBoyland & @SimonCapewell99<br>@DrAseemMalhotra supports<br>#Obesity #Prevention #ProtectOurKids #Sugar #Salt	*total*=51<br>*retweets*=15<br>*replies*=0<br>*likes*=32<br>*quotes*=4

Stanford University (StanfordMed)	February 19, 2016	Prof. John Ioannidis sheds some light on a new study that finds mixed evidence that salt is bad for you:	*total*=46<br>*retweets*=16<br>*replies*=4<br>*likes*=26<br>*quotes*=0
Tom Frieden (DrTomFrieden)	October 15, 2021	Excess salt intake will cause an estimated 1.6 million deaths worldwide this year alone. We face an epidemic of preventable disease from unhealthy food. Reducing sodium consumption can save millions of lives.	*total*=1941<br>*retweets*=322<br>*replies*=90<br>*likes*=1505<br>*quotes* =24
University College London (BscPopHealth)	June 8, 2017	Nothing quite like election night with a packet of salt + vinegar squares and a can of irn bru! Gordon, Comms @bscpophealth	*total*=4<br>*retweets*=1<br>*replies*=0<br>*likes*=3<br>*quotes* =0
Food and Agriculture Organization of the United Nations (FAO)	October 1, 2019	5 eating habits that are good for us 👇<br>🍅Eat plenty of veggies & fruits<br>🍔Watch your intake of fats<br>🍩 Cut back on sugar<br>🍽️Reduce salt<br>💧 Drink lots of water<br>#WorldFoodDay #healthydiets	*total*=1306<br>*retweets*=438<br>*replies*=14<br>*likes*=838<br>*quotes*=16
United States Department of Agriculture (USDA)	February 4, 2022	The new final rule – Child Nutrition Programs: Transitional Standards for Milk, Whole Grains, and Sodium – establishes the following requirements beginning School Year 2022-2023 ⬇️	*total*=118<br>*retweets*=31<br>*replies*=20<br>*likes*=53<br>*quotes*=14
World Action on Salt (actiononsalt)	March 2, 2016	Salt is the easiest of things to reduce—it's often there just for flavor or preservation. Let's get on with it! #SaltAwarenessWeek	*total*=148<br>*retweets*=9<br>*replies*=52<br>*likes*=8<br>*quotes*=79
World Heart Federation (worldheartfed)	December 22, 2021	Most people don't think about lowering their salt intake until it's too late. But did you know that reducing the sodium in your diet is one of the most effective ways to control your blood pressure & reduce your risk of #CVD 💔 ? This holiday season, #UseHeart to #SeasonWithSense.	*total*=38<br>*retweets*=10<br>*replies*=0<br>*likes*=26<br>*quotes*=2
World Health Organization (WHO)	May 6, 2021	Most people consume 2x the WHO-recommended 5 g of daily #salt intake, putting themselves at greater risk of the heart diseases & strokes that kill an estimated 3 million people each year.<br>🆕 WHO benchmarks help countries reduce salt intake & save lives	*total*=2281<br>*retweets*=651<br>*replies*=53<br>*likes*=1507<br>*quotes*=70

Abbreviations: RT = retweet; NCDs = noncommunicable diseases; US = United States; CVD = Cardiovascular disease; WHO = World Health Organization; FDA = Food and Drug Administration; NEJM = The New England Journal of Medicine
